# Assessment of resources for physical activity and understanding people’s perception and practices regarding physical activity in an Indian city

**DOI:** 10.1186/s12889-023-16846-7

**Published:** 2023-10-11

**Authors:** Susheel Gautam, Kruthika B N, Akashanand, Aaheli Roy, Pradeep S. Banandur, Arvind Anniappan Banavaram

**Affiliations:** 1 Department of Epidemiology, National Institute of Mental Health and Neurosciences, Bengaluru, 560029 India; 2 JSS School of Public Health, Mysuru, 570015 India

**Keywords:** Kolar, Physical activity, Resources, Perception, Practices

## Abstract

**Background:**

Promoting physical activity in urban India is imperative considering the burden of non-communicable diseases in the country. Planning for improving population level physical activity needs sound understanding of availability and quality of resources/facilities for physical activity and knowing people’s perception and practices regarding the physical activity.

**Methods:**

A cross-sectional study was undertaken in Kolar city of Karnataka state in India. All the resources/facilities required for supporting physical activity were mapped and their quality was assessed utilizing adapted version of physical activity resource assessment questionnaire. The information regarding latitude, longitude and approximate size of the resource was obtained using a hand-held GPS tracker. 495 individuals aged ≥ 18 years, selected by two stage cluster random sampling with probability proportionate to population size technique, were interviewed to assess their perception and practices regarding physical activity using semi-structured questionnaire and global physical activity questionnaire.

**Results:**

Kolar city has 36.3 physical activity resources per lakh population and per person availability of park and playground area was 0.4 Sq. meters. Available resources were concentrated in the center of the city. Half of the sports facilities and 14 of the 17 recreational facilities in the city were of poor to mediocre quality. 38.2% of adults in Kolar city were found to be physically active. Only 19.2% of the study participants had accessed sports/fitness facilities/playgrounds in past 3 months and only 18.8% of the study participants accessed parks in the previous 3 months. 28.6% to 59.1% of the participants preferred ‘walking’ for work, college and shopping. Less than 5% of the participants preferred and used cycle as a mode of transport. 1/3^rd^ of the study participants felt that Kolar city is safe of walking and 44.6% felt that the city is safe for cycling.

**Conclusion:**

Creating enabling environment by increasing the number and quality of resources/facilities for physical activity along with their equitable distribution is required to promote and improve population level physical activity in Kolar city. Urban planning with a focus on non-motorized transport including walking would contribute to improved people’s perception and practices regarding physical activity in the city.

**Supplementary Information:**

The online version contains supplementary material available at 10.1186/s12889-023-16846-7.

## Background

Physical inactivity is one of the major risk factors for non-communicable diseases (NCDs) and premature deaths globally. The risk of developing cardiovascular illnesses, diabetes, and cancer is 20–30% higher for people who are not physically active enough compared to those who are. Regular exercise is known to promote mental health and reduces the risk of developing depression The spectrum of health benefits of physical activity spans across the lifespan including children (improves cognitive functioning and academic performance) and the elderly (reduces chronic conditions and cognitive deterioration) [1, 2]. Overall physical inactivity shortens one’s life span by 3–4 years [2].

Despite evidences showing clear benefits of physical activity, the prevalence of physical inactivity is very high in India. According to the recent National Noncommunicable Disease Monitoring Survey (NNMS) report (2017–2018), 54.5% of Indians did not engage in the recommended amount of physical activity [3]. Generally, physical inactivity in urban setting is more when compared to rural area (40.6%) and in urban area (63.3%) [4]. In urban India, more than half of women (60.2%) were found to be insufficiently physically active, compared to (44.2%) of men [5].

The WHO’s global action plan on physical activity (2018–2030) emphasizes systems-based approach for increasing physical activity in the population. It stresses on creating active societies, active environment, active people and active systems. Accordingly, the interventions should focus on community-based campaigns, capacity building of all stakeholders, regular population surveillance of physical activity, interventions for improving road safety and the personal safety of pedestrians, cyclists, etc. strengthen access to good quality public and green open spaces, green networks, recreational spaces and sports amenities by all people among others [6].

The need of the hour is to improve physical activity across cities of India which would enable the country to achieve the goals and targets set under the national action and monitoring framework for prevention and control of NCD in India [7]. To implement evidence-based interventions for increasing physical activity in cities, based on a systems approach, the need for understating the prevailing ecosystem for the same is essential. Unfortunately, studies in India have predominately focussed on assessing the prevalence of physical activity and neglected other determining forces of physical activity. Furthermore, cities are varied and diverse which underlines the importance of cities specific data for planning, implementing, and evaluating evidence-based interventions for promoting physical activity at the city level [6]. Hence the present study was undertaken in Kolar city of Karnataka with the objectives of i) Assessment of the availability and quality of resource/facilities for physical activity in Kolar city and ii) assessment of the perception and practices pertaining to physical activity among adults in Kolar city.

## Methods

The present cross-sectional study was undertaken in Kolar city of Karnataka during 2019. The city is located on the southern plains of Karnataka with a total area of 46.56 km sq and has a population of 1,38,462 according to 2011 census [8]. The morphological pattern of Kolar city, which is a small city, is similar to other cities in India. It has mixed pattern of development (residential and commercial localities co-exist) with unplanned urbanization and population density decreasing from the center to the periphery. The city has mixed road users and the roads are overcrowded with pedestrians, bicycle riders, two wheelers, cars and heavy vehicles.

### Study population

Resources and sports facilities supporting physical activity and situated within the administrative limits of Kolar city municipal cooperation were the study units. Resources considered in the study include: stadiums, parks, playgrounds, fitness centers, yoga centers, individual sports facility, and sports clubs. Sports facilities considered in the study were; basketball court, volley ball court, tennis court, badminton court, football ground, cricket ground, swimming pool, walking trail/ path, wrestling, running track, gymnasium, exercise station/ open gym, garady mane/ taleem/ akhada, table tennis court, kho-kho ground, kabaddi ground, ball badminton ground. Resources and sports facilities available within the educational institutions in the city but with restricted access to general population were excluded. The study also aimed to assess the perception and practice regarding physical activity among adults in Kolar city. For this, all adults aged 18 years and above and who are usual residents of the city were considered as the study population.

### Sample size and sampling technique

All the resources for physical activity and sports facilities in Kolar city were included for assessing the availability and quality of resources/facilities. These resources and sports facilities were identified by conducting a walkthrough survey in Kolar city and also by snowball sampling technique. For assessing the perception and practices of adults in Kolar regarding physical activity the estimated sample size was 521. The estimated prevalence of physical inactivity among adults (above 18 years) in urban area of south India varied between 71% and 79%. Considering the lower prevalence estimate of 71%, at 95% confidence level, design effect of 1.5 and non- response rate of 10%, the estimated minimum sample size is 521.

Participants for the study were selected by two stage cluster random sampling with probability proportionate to population size (PPS) sampling technique. In the first stage, wards were selected through PPS method and in the next stage one CEB was selected by Simple random sampling from within the selected ward. Census Enumeration Block (CEB) were defined as cluster. It was decided to interview 30 adults per cluster (i.e., CEB). The rationale for deciding 30 adults per cluster was that it would lead to selection of 18 wards (to cover 521 adults) and thereby survey would cover 50% of the wards (i.e., 18 out of 35 wards) in Kolar city.

Within the selected cluster the center of the cluster was identified and all streets converging at the center were serially numbered. Following this, one of the streets was selected randomly using currency methods. Within the selected street households were selected using systematic random sampling technique with the random selection of the first household. All the adults (≥ 18 years) in the selected households and who were usual residents of the given locality were considered eligible for the survey. All the eligible participants available in house at the time of first visit were requested to participate in the study and those consenting were interviewed. Individuals refusing to participate in the study were considered as non-responders. Individuals not available at the time of first visit were contacted at a day and time convenient to the participant which was decided after discussing with the participant. Three visits were planned for the selected household and those individuals not available even after 3^rd^ visit were considered as non-responders.

### Study instrument

“The Physical Activity Resource Assessment (PARA) instrument was suitably adapted for the present study [9]. The PARA questionnaire was modified to suit the local needs with a focus on functionality/utility of the facilities. Following parameters were assessed for each facility: approximate size, capacity (if indoor), cost of use, hours of operation, features available in the facility, amenities present and incivilities observed within the facility. These parameters were assessed objectively and the tool was finalized after the pilot study.

We derived a ‘usability score’ as an objective measure for assessing the quality of the resource. This score was based on a) availability of displayed information (Display of information of functioning hours, do’s/ don’ts and amenities available), b) presence and quality of amenities (includes fencing, lighting, benches, shelters, bathrooms/shower facility, toilet, locker/locker room, trash container, drinking water facility) and c) presence and degree of incivilities (includes presence of unattended animals, evidence of alcohol use, auditory annoyance, litter / refuse, overgrown grass/shrubs) in the resource. If the information was displayed (functioning hours, do’s/ don’ts and amenities available) then ‘1’ mark was assigned for each parameter and ‘0’ was assigned otherwise. Amenities were scored according to their quality as ‘0 = Not Present, 1 = Poor, 2 = Mediocre and 3 = Good’. For calculating ‘usability score’ only those amenities which were essential for a resource were included. For example, a) Stadiums and playgrounds were assessed for all amenities, b) parks were assessed for all amenities (mentioned above) except ‘bathrooms and locker rooms’, c) all indoor resources like fitness centers and yoga centers were assessed for all amenities (mentioned above) except fencing, shelter, animals unattended and overgrown grass/ shrubs. The incivilities were scored for animals unattended and evidence of alcohol use as ‘present = 1 and absent = 0’. The other incivilities (auditory annoyance, litter/refuse and overgrown grass/ shrubs) were scored as ‘0 = Not Present, 1 = Little/Few, 2 = Some and 3 = A lot’. Then these incivility scores were reversed to calculate usability score. For animals unattended and evidence of alcohol use ‘0 was converted to 1’ and ‘1 was converted to 0’. For the other incivilities (auditory annoyance, litter/refuse and overgrown grass/ shrubs) ‘0 was converted to 3’, ‘1 was converted to 2’, ‘2 was converted to 1’ and ‘3 was converted to 0’. Now the usability score was calculated as described below:

The formula used for calculating usability score:

$$\mathrm{Usability\;score }= \frac{\left(\mathrm{Scores\;of\;displayed\;information }+\mathrm{scores\;of\;amenities }+\mathrm{reverse\;scores\;of\;incivilities}\right)}{\mathrm{Culmulative\;maximum\;scores}} \times 100$$

The quality of the sports facilities available within the city was assessed and categorized as poor, mediocre or good based on set of observations (Supplementary table 1). The information regarding latitude, longitude and approximate size of the resource was collected using a hand-held GPS tracker (GARMIN etrex 10). After identifying the resources, the field investigator stood in front of the resources and captured the latitude and longitude of the resources using a special handheld GPS tracking device (GARMIN etrex 10). This was done to spatially locate the resources on GIS map. For assessing the area of resources particular for parks and playgrounds, the field investigator identified one start point for the resources then switched on the GPS tracker and went around the resource area to finally come back to the original start point. Using this method, the area of the parks and playgrounds were assessed. The prevalence of physical activity among the study participant was assessed using Global Physical Activity Questionnaire (GPAQ) developed by World Health Organization [10]. Insufficient physical activity is defined as those achieving less than 150 min of moderate-intensity physical activity per week or less than 75 min of vigorous-intensity physical activity per week or an equivalent combination of moderate- and vigorous-intensity physical activity less than 600 metabolic equivalent minutes per week [11]. Knowledge and perception of the study participants regarding various aspects of physical activity was assessed using a semi structured questionnaire. All the data in the present study was collected by both observation and interview of the key personnel/ study participants as appropriate.

### Data management & statistical analysis

Quantitative data was summarized into means and standard deviations and Qualitative data was summarized into frequency and percentages. The prevalence of insufficient physical activity was assessed for the overall study population and by various socio-demographic characteristics including household income quintile.

## Results

**Fig. 1:**
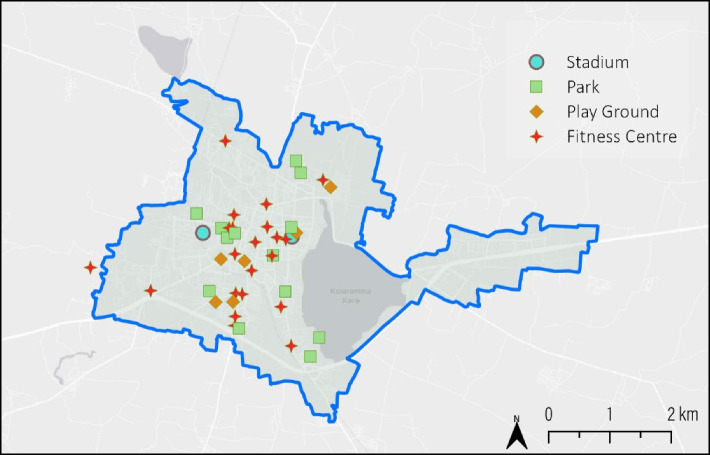
Spatial distribution of parks, playgrounds, stadiums and fitness centers in Kolar city

The availability of the resources is an important factor influencing the level of physical activity in a community. In Kolar city, there are 36.3 physical activity resources per lakh population. All the stadiums, parks, and playgrounds in the city were publicly owned. Per person availability of park and playground area was estimated to be 0.4 sq. meters in the city which was very low as compared to 3 sq. meters per person which is the minimum norm suggested by NBC (National Building Code) [12]. Spatial mapping of parks and playgrounds show that most of them are situated in the center of the city. The new developing areas and peripheral areas of city lack most of the resources (Fig. 1).

All the stadiums and most of the fitness centers in the city had a usability score of 50% and above. However, parks and playgrounds in the city had low usability score with all of them scoring below 50% (Table 1). Affordability of resources is very important to keep them in reach of general public. All the stadiums, parks and playgrounds, most of the yoga centers (4 out of 6) and majority of the sport club (10 out of 12) did not charge any user fee. Though stadiums did not charge user fee, they had user fee for select facilities (user fee for gymnastic facility). Interestingly 10 of the 13 registered sports facility in the city were free for public participation.

**Table 1:** Usability score of the resources available for performing physical activity in Kolar City

SI.No	Facility(n)	< 25%	25%—< 50%	50%—< 75%	≥75%
1	Stadiums (*n* = 2)	0	0	0	2
2	Parks (*n* = 13)	3	10	0	0
3	Playgrounds (*n* = 6)	5	1	0	0
4	Fitness centers (*n* = 13)	0	1	5	7
5	Yoga center (*n* = 8)	0	3	5	0
6	Individual Sports Facility (*n* = 1)	1	0	0	0
7	Sports club (*n* = 1)	0	0	1	0

There were a total of 58 sports, fitness and recreational facilities in the city. Half of the sports facilities in the study area were of poor to mediocre quality and most of the recreational facilities in the city (14 out of 17) were of poor to mediocre quality (Table 2). Most of the available facilities (63.6%) in Kolar city for promoting physical activity did not charge any user fee i.e., access to them were free of cost. Only fitness centre (all 13), few yoga centres (2 out of 8) and few sports club (2 out of 13) charged some amount for utilizing its services (Supplementary table 2).

**Table 2:** Availability and quality of the sports, fitness, recreational facilities present in select resources in Kolar city

Sl. No	Nature of facility (n) (Nature of ownership)	Poor	Mediocre	Good
**Sports Facilities**				
1	Basketball Court (*n* = 3) (Public = 3)	0	1	2
2	Volley ball court (*n* = 4) (Public = 3, Private = 1)	1	2	1
3	Badminton court (*n* = 1)	0	0	1
4	Football Ground (*n*- = 1) (Public = 1)	0	1	0
5	Cricket ground(*n* = 1) ( Private = 1)	0	0	1
6	Running track (*n* = 1) (Public = 1)	0	1	0
7	Table tennis court (*n* = 2) (Public = 2)	0	0	2
8	Kho-Kho ground (*n* = 2) (Public = 2)	0	0	2
9	Wrestling ground (*n* = 1) (Public = 1)	0	1	0
10	Kabaddi ground (*n* = 1) (Public = 1)	0	1	0
11	Ball badminton ground (*n* = 1) (Public = 1)	0	1	0
	**Total (*n* = 18)**	**1**	**8**	**9**
**Fitness Facilities**				
12	Gymnasium(*n* = 11) (Public = 1, private = 10)	0	0	11
13	Exercise station(*n* = 1) (Public = 1)	0	0	1
14	Yoga centers (*n* = 8) (Private = 8)	0	0	8
15	Garady mane( *n* = 2) (Public = 1, private = 1)	0	0	2
16	Aerobics center(*n* = 1) (Private = 1)	0	0	1
	**Total (*n* = 23)**	**0**	**0**	**23**
**Recreational Facilities**				
17	Walking trail/ path(*n* = 10) (Public = 10)	2	6	2
18	Play equipment’s(*n* = 7) (Public = 6, private = 1)	2	4	1
	**Total (*n* = 17)**	**4**	**10**	**3**

**(**Garady mane – traditional gym)

We interviewed 495 individuals in the city (males 45.7%, females 54.3%). Most of the participants felt that physical activity reduces individual’s risk of developing obesity (83.2%) and stress (82.8%). Considerable proportion of the population felt that either physical activity does not reduce the risk or they don’t know about the benefits of physical activity with respect to hypertension (36.4%), diabetes (37.8%), heart attack (47.1%), depression (49.9%) and stroke (58.0%). 24% of study participants expressed that physical activity can reduce the risk of colon and breast cancer. Interestingly 97.6% of study participants said that one should spend 30 min or more doing physical activity in typical day. Two third of the study population (66.8%) felt that they are fairly physically active and almost a third (30.8%) felt that they are very physically active. Although majority (97.6%) felt that they are physically active, 38.2% of study participants had less than recommended amount of physical activity (Table 3). The proportion of people participating in vigorous or moderate intensity sports, fitness or recreational activity was very low (21%). The estimated median sedentary time in the study population was 6 h (IQR:4 TO 10 h).

**Table 3:** Prevalence of insufficient physical activity (< 600 MET minutes per week) among adults (≥ 18 years) in Kolar city (*n* = 495)

	Frequency	Percent
Proportion of population with insufficient physical activity (< 600 MET minutes per week)	189	38.2 (95% CI = ± 4.3)
Proportion of population whose work involve vigorous intensity activity for at least 10 min continuously	28	5.7
Proportion of population whose work involve moderate intensity activity for at least 10 min continuously	190	38.4
Proportion of population who walk or bicycle for at least 10 min continuously	254	51.3
Proportion of population who participate in vigorous intensity sports, fitness or recreational activity for at least 10 min continuously	24	4.8
Proportion of population who participate in moderate intensity sports, fitness or recreational activity for at least 10 min continuously	80	16.2

Most of the participants felt that the facilities available for physical activity is ‘either poor or very poor’ in their neighbourhood (71.9%) and in Kolar city (49.7%). It was noted that only 19.2% of the study participants had visited sports, fitness facilities or playgrounds in past 3 months and only 18.8% of the study participants visited parks in the previous 3 months.

The common reasons for people not engaging in sports, fitness and recreational facilities were lack of time (55.4%), lack of interest (28.8%), ‘does not have friends/ neighbors to accompany’ (14.3%), ‘no/ poor availability of sports or fitness facilities in neighborhood’ (13.1%), medical issues (9.4%) and cost (2.2%).

**Fig. 2:**
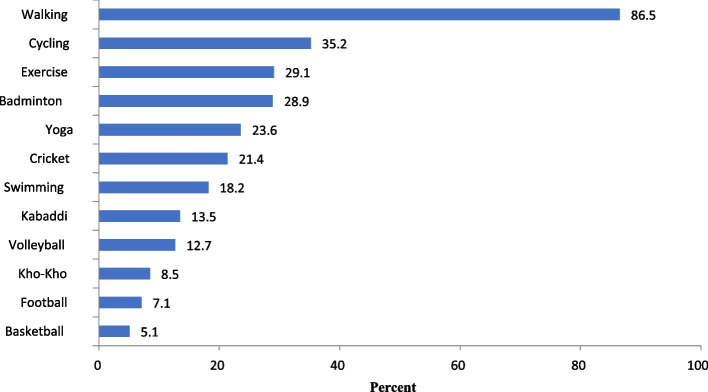
Distribution of study population by their preferred choice of leisure related physical activity

There is need to promote leisure related physical activity in Kolar city for which understanding the people’s preference is very important. Figure 2 shows preferred choice of leisure related physical activity in Kolar city. The majority of the study participants (86.5%) preferred walking as their leisure related physical activity.

Majority of the participants preferred using their ‘own motor vehicle’ (42.8–63.8%) as mode of transport for various purposes (going to work, college, shopping, social or recreational). Interestingly 28.6%—59.1% of the participants preferred ‘walking’ as a mode of transport for work, college and shopping. Similar picture is observed regarding the actual mode of transport also where majority of the participants used their ‘own motor vehicle’ (28.6- 57.7%) and ‘walking’ (9.9–40.0%) as their main modes of transport within above stated domains (Table 4). Only one third of the participants felt that Kolar city is safe of walking and less than half (44.6%) felt that the city is safe for cycling.

**Table 4:** Distribution of study population by their preferred and actual modes of transport

	Walking (%)	Cycling (%)	Public transport (%)	Intermediate public transport (%)	Own motor vehicle (%)
**Distribution of study population by their preferred mode of transport**					
Work (*n* = 215)	82(38.1)	8(3.7)	19 (8.8)	10 (4.6)	96 (44.6)
College (*n* = 28)	8 (28.6)	0 (0)	5 (17.8)	3 (10.7)	12 (42.8)
Shopping (*n* = 464)	274(59.1)	5(1.1)	5(1.1)	13 (2.8)	167(35.9)
Social/recreational purpose (*n* = 494)	46 (9.3)	1(0.2)	68(13.8)	64 (12.9)	315 (63.8)
**Distribution of study population by the actual mode of transport**					
Work (*n* = 215)	86(40.0)	9(4.2)	21 (9.8)	10 (4.7)	89 (41.4)
College (*n* = 28)	8 (28.6)	0 (0)	9 (32.1)	3 (10.7)	8 (28.6)
Shopping (*n* = 464)	289(62.3)	4 0.9)	6 (1.3)	15 (3.2)	150(32.3)
Social/recreational purpose (*n* = 494)	49 (9.9)	1(0.2)	87(17.6)	72 (14.6)	285 (57.7)

When we consulted people about possible suggestions for promoting walking and cycling in Kolar city, we were surprised to see that people gave wide range of suggestions like doing awareness programs, improving physical activity facilities (Ensuring availability of well-maintained parks nearer to their houses), improving infrastructure for walking, cycling and other sports facilities and some legislative measures like increasing the fuel prices (Table 5).

**Table 5:** Peoples’ suggestions to promote walking, cycling and sports in Kolar City

Walking	Cycling	Sports
1) Awareness program to educate and motivate people about the benefits of walking 2) Ensuring availability of well-maintained parks nearer to their houses 3) Safety along the access roads to the parks (e.g., roads with adequate street lights) 4) Expressed need for separate and safe places (e.g., parks) exclusively for women 5) Improve footpaths, road condition and implement measures to manage traffic 6) Reduce dust and pollution in the air by improving road condition, reduction in traffic, planting more trees in the city 7) Few suggested increasing fuel (diesel/ petrol) prices so people avoid using their own vehicle and use walking, cycling or public transport as their commute 8) Some suggested formation of walking clubs to promote walking	1) Awareness program to educate and motivate people about the benefits of cycling 2) Improving road conditions to make them safe and suitable for cycling 3) Many suggested the need for safe and separate pathways/ lanes/ roads/ places for cycling 4) Some suggested formation of cycling clubs 5) Cycles to be made available at cheaper price	1) Awareness program to educate and motivate people about the benefits of sports 2) Sports should be mandatory in schools and colleges 3) Measures to reduce the use of mobile phones and utilize that time for sports /recreational activity 4) Leveraging children and educational institutes to promote sports in families and communities 5) Increasing the number of facilities and proper maintenance of the existing facilities 6) Safety along the access roads to the stadiums (e.g., roads with adequate street lights) 7) Expressed need for separate and safe sports facilities exclusively for women 8) Ensuring availability of free coaching services for various sports 9) Increase the number and type of sports events/tournaments in the city 10) To promote sports with the help of local sports personalities/professional players 11) Employers should provide free time and facilitate sports within the organization 12) Improve the economic condition so that people can spare time for sports instead of spending all their time for earning bread and butter

## Discussion

To the best of our knowledge, this is the first comprehensive study in India which simultaneously examined the prevalence of physical activity, availability and quality of resources for physical activity and explored perception of people regarding physical activity at the city level. The major highlights of the study are, there are 36.3 resources and facilities per lakh people supporting physical activity in Kolar city. The total area of park and playgrounds in the city was 0.4 sq. meters per person and most of them were located in the core area of the city. All the parks and playgrounds in the city had low usability scores (< 50%), majority of the leisure facilities (like walking trail and play equipment in parks and playgrounds) and 50% of the sports facilities in the city were of poor to mediocre quality. Around 40% of the adults in the city were physically inactive and the proportion of the people engaging in leisure, exercise or sporting activities was very low. Nearly 3/4^th^ and half of the study participants expressed that the availability of the facilities for physical activity in their neighborhood and in Kolar city as either poor or very poor respectively. Since most of facilities were free of cost, we assume cost is not a barrier to utilization of these resources.

Availability of parks, playgrounds and other sports and fitness related resources/facilities is vital for promoting physical activity in urban area. Parks play a vital role in facilitating physical activity and have become widely recognized as important resource for community health promotion [13]. James F Sallis et al. observed that Number of parks available in the neighborhood is significantly associated with physical activity among the residents in the neighborhood (odds ratio = 1.16) [14]. In Kolar city there were 36.3 physical activity resources for one lakh population including 13 parks and 6 playgrounds. Kolar city has 8.7 parks per 1 lakh population. The city of Bangalore in Karnataka has 1146 parks [15] which comes out to be 13.5 parks for every 1 lakh population which is better than in Kolar city. The number of parks across different cities in India ranged between 525 parks in Chennai [16] (2.1 parks per 1 lakh population) to 18000 parks in Delhi (164 per 1 lakh population) [17]. Cities of the US consists of parks ranging from 210 in Fort Worth (25 per 1 lakh population) and 1678 in New York (24 per 1 lakh population) [18].

Per person availability of park and playground area is relatively a better indicator than number of parks. In Kolar city per person availability of the park and playground was 0.4 sq. meters. The availability of open space (park and playground) in city of Bangalore in Karnataka is 2.2 sq. meters per person [19]. The per capita availability of green space (including parks and playgrounds) across different cities in India ranged between 0.46 sq. meters in Chennai [20] to 20 sq. meters in Delhi [21]. The recommended per-capita green space according to UDPFI (Urban Development Plans Formulation and Implementation) is 10–12 sq. meters [12]. This clearly shows that the availability of parks and playgrounds in Kolar city is highly inadequate. Evidence shows that the total area of green space, the size of open space, and entertainment facilities in the neighborhood are significantly correlated to residents’ physical activity. Basketball courts, volleyball courts, swimming pools, and sports equipment in the neighborhood is also likely to promote physical activity [22].

The spatial distribution of parks influences physical activity among the residents. Most of the parks and playgrounds in Kolar city are located in the central part of the city and the peripheral or newly developing areas do not have parks and playgrounds. A study by Ariane reports that exercise facilities, including parks, that are conveniently located in the community are significantly associated with vigorous physical activity among both adults and children [23]. Residents living within 0.5 miles of the park reported leisurely exercising 5 or more times per week more often than those living more than 1 mile away (49% vs 35%; *p* < 0.01) [23]. Overall, the availability of number of parks, per capita availability of parks and playgrounds area and their spatial distribution in Kolar city could influence the physical activity level among adults in the city.

National Park and Recreation Association of United States of America reported that the presence of active park features and support is linked with higher park use levels and higher moderate-to-vigorous physical activity [24]. Several other studies also have observed the quality of facilities and amenities in the park to be significantly associated with increase in park visitation and increase in physical activity level in the neighborhood [25, 26]. In the present study, the quality of stadiums and fitness centers were good which is indicated by their high usability score. Unfortunately, all the parks and playgrounds in the city scored poorly indicating poor quality of the facilities. Amenities like proper fencing, lighting, benches, shelters, bathrooms, toilets, locker facility, trash container and drinking water facility were either not available or were very poorly managed. Incivilities like unattended animals, auditory annoyance, litter or refuse and overgrown grass or shrubs were found in a considerable number of studied parks and playgrounds. Similar observation was made by a study conducted in Bangalore according to which only 4–6% parks and playgrounds had public toilet facilities, 1–3% had drinking water facilities, 56% had lighting facilities, 77% had seating area [27]. A study assessing public parks in Delhi (capital city of India) reported that only 11% had toilets and only 44% of the public parks surveyed had recreational facilities for children [28]. From this, it can be inferred that parks and playgrounds have not received adequate attention from the policy-makers, administrators and politicians both in the country and in the study area. All the concerned stakeholders should recognize parks, playgrounds and other sports facilities as public good and appreciate their role in promoting physical activity in the population along with other potential health benefits. In the light of the available evidence, the Kolar city administration should not only focus on increasing the number of recreation and sports facilities in the city but should equally focus on providing adequate amenities and enhancing the quality of those facilities.

Prevalence of physical inactivity in our study is 38.2%, this is similar to studies done in India (54.4%, 52.1%, 63.3%) [29–31]. If we look at the pattern of physical inactivity, proportion of people whose work involves vigorous intensity activity is very low (5.7%). Considerable proportion of people’s work involve moderate intensive physical activity (38.4%) and half of study population are engaged in walking and bicycling for at least 10 min continuously which contributes to overall physical activity. The proportion of the population for whom sports, fitness and recreational activity contributes to overall physical activity is very low (20%). Interventions aimed at promoting physical activity in the population should have a comprehensive approach. In the study population focusing on increasing people’s participation in sports, fitness and recreational activities can improve the physical activity level in the population. Though overall prevalence of physical inactivity is reported in this study, for better planning of interventions and addressing inequities in physical activities data across various socio-demographic groups is preferred. This is a limitation, as the sample size in the present study was estimated considering only one stratum i.e., adults aged ≥ 18 years in Kolar city and this sample size does not provide reliable prevalence estimates by sex, age group and other socio-demographic strata. The findings from this study could also be influenced by the response and recall bias associated with using GPAQ [32]. However, GPAQ being a validated and recommended tool by WHO-STEPS survey, the biases are unlikely to have a major influence on the prevalence estimates of the study [33].

According to health education theories perception about availability and quality of the facilities plays an important role in promoting individual’s physical activity. In our study perception about quality of facilities is low and the proportion of people using facilities is also low. 13.1% expressed that unavailability of facilities was the reason for not participating in sports or fitness-related activities A study by Duncan et al. reports that perceptions of neighborhood cleanliness and footpath condition showed associations with the likelihood of achieving sufficient levels of physical activity [34].The study conducted by Marui et al. reports that 36.8% and 35.2% of the subjects were inactive or did not reach 150 min/week on transport-related physical activity, respectively. The common reasons cited for the same were the absence of parks and athletic courts, presence of garbage, absence of street lighting were associated with lower levels of commuting PA (Marui W corseuil) [35]. So, Kolar city administration should bring the overall changes in the system to improve the facilities and also improve the people’s perception which will lead to better physical activity at the population level. Planning an intervention along with public participation or community participation is essential. In light of this, it is likely that individuals will use services more frequently when their opinions and suggestions are taken into account while developing policies and programmes. In that directions people’s choice and preferences have been elucidated which is available in Table 5. Whenever the Kolar city administration wants to plan of improving the physical activity resources then these preferences should be considered.

Active community, including cycling and walking is not only associated with increase in the level of physical activity but also yields several other health benefits [36, 37]. Walking for 30 min or cycling for 20 min on most days reduces the mortality risk by at least 10%. Active commuting is associated with about 10% and 30% decrease in the risk of developing cardiovascular disease and type 2 diabetes respectively [36]. Cycling for transportation and higher levels of overall non-exercise physical activity are associated with 35% reduction in risk for all-cause mortality. (Charles Mattews) [38]. In the present study, use of own vehicle and walking were the most common preferred and used mode of transport for various purposes. Less than 5% of the participants preferred and used cycle as a mode of transport. Lack of time, more distances to travel, convenience, unsafe roads, unavailability of separate pathways/ footpath for walking and cycling, and owning a vehicle as a status symbol could have led to increased motorization and decreased the preference for active commuting in the study population.

## Conclusion

Non-Communicable Diseases are a major public health problem in India and particularly in urban India. Level of physical activity, an important risk factor of NCDs, is generally low in urban areas. Present study reveals that adults residing in Kolar city of India are less physically active. Study also highlights that the quantity and quality of the resources available in the city required for supporting physical activity is highly inadequate and inequitably distributed. Need exists to support and strengthen active commuting in the population. NCDs demand multisectoral intervention and in that context, Department of urban planning and department of transport in the state and Kolar city could utilise the available evidence from this study to improve the urban infrastructure including parks, play grounds and other sports and physical activity facilities for increasing the level of physical activity in the population and reducing the health burden associated with them.

### Supplementary Information

**Additional file 1:** **Supplementary Table 1.** Operational definitions used to assess the quality of the facilities. **Supplementary Table 2.** Prevalence of insufficient physical activity by socio-demographic characteristics of the study population.

## Data Availability

The datasets used and analyzed during the current study are available from the corresponding author on reasonable request.

## Abbreviations

NIMHANS: National Institute of Mental Health and Neuro Sciences
NCD: Non-Communicable Disease
NNMS: National Noncommunicable Disease Monitoring Survey
PPS: Proportionate to Population Size
WHO: World Health Organization
CEB: Census Enumeration Block
PARA: Physical Activity Resource Assessment
GPAQ: Global Physical Activity Questionnaire
NBC: National Building Code
UDPFI: Urban Development Plans Formulation and Implementation
