# Supplementary material for: Assessment of resources for physical activity and understanding people’s perception and practices regarding physical activity in an Indian city
Source: BMC Public Health. 2023 Oct 11;23:1969. doi: 10.1186/s12889-023-16846-7 (PMC10568767; doi:10.1186/s12889-023-16846-7)
Supplement: Supplementary file 1 — Additional file 1: Supplementary Table 1. Operational definitions used to assess the quality of the facilities. Supplementary Table 2. Prevalence of insufficient physical activity by socio-demographic characteristics of the study population. [file 12889_2023_16846_MOESM1_ESM.docx]

**Supplementary Tables**

**Supplementary Table 1. Operational definitions used to assess the quality of the facilities**

| **Feature** | | **Poor** | **Mediocre** | | **Good** | |
| --- | --- | --- | --- | --- | --- | --- |
| **Baseball field –** | | Surface of fields is uneven, unsafe, no overhead lighting, no benches for players, fencing in poor condition or nonexistent | Surface of fields is uneven, slightly unsafe, no overhead lighting, + benches for dugouts. Some fencing existent, but not 100% intact | | Surface of fields is uniform, no rocks/barriers to running bases, have overhead lighting, + benches for dugouts. Have bleachers for spectators, intact backstop fencing | |
| **Basketball courts –**  **(BB courts)** | | Court or hoop is in very bad condition, almost unstable | Hoop is missing a net, rim is bent, court has cracks or weeds | | Hoop is straight and has a net or chain, court is playable | |
| **VB courts** – | | Playing surface has debris or cracks or bumps all over, net is almost unusable or missing | Playing surface has some debris or cracks or has 1 – 5 bumps, net is sagging or has holes | | Playing surface is free of debris and smooth, net is in good condition | |
| **Cricket field** | | Grass coverage may be poor in 50% or > of the field, rough surface, hazards and/or trash on the field, pitch is not identifiable | Grass coverage may be sparse in a few places, grass may be too high, some trash or debris on field, pitch is identifiable but uneven surface | | Field has uniform grass coverage and is well-mowed, no trash or debris on field; nets, if furnished, are intact; pitch is identifiable with even surface | |
| **Football/ hockey fields –** | | Grass coverage may be poor in 50% or > of the field, rough surface, hazards and/or trash on the field | Grass coverage may be sparse in a few places, grass may be too high, some trash or debris on field | | Field has uniform grass coverage and is well-mowed, no trash or debris on field; nets, if furnished, are intact. | |
| **Gymnasium / Garady mane / akhada /Exercise Stations / Open gym/ Play equipment (describe if different than traditional play equipment – slide, swings, monkey bars) /Yoga center** | | Several pieces are in need of major repair and is almost or unstable, there is a lot of trash, and the ground is overgrown or barren | Some equipment is in need of minor repair, there is some trash, and the ground needs some improvement | | In good condition, variety of pieces, ground in good condition, well-kept and clean | |
| **Swimming pool** | | Swimming pool has very discolored water or too little water, surrounding surface is in need of repair, trash in or around pool – not safe for use | Swimming pool or deck needs minor cleaning or treatment | | Swimming pool is clean, well-lit. surrounding surface is safe as well as exit/entry points | |
| **Walking track** | | Sidewalk has major damage and needs repair, almost unusable | Sidewalk has some debris, cracks or uneven surfaces, but otherwise usable | | Sidewalk is smooth, clear of debris | |
| **Trails – running/biking/ Skating court** | | Surface is unsafe in many places, there is a lot of debris, no signage about appropriate use | Surface is in places uneven or in need of minor repair, may be a few hazards or avoidable debris | | Surface is smooth, without unmarked hazards or debris , has signage re: appropriate users | |
| **Tennis/ badminton courts** | | Courts have cracked surface, nets are in major need of repair, debris is evident; almost unusable | Court surface and nets are in need of some repair, but otherwise usable | | Tennis court surface and nets are in fairly good condition | |
| **Amenities** | **Poor** | **Mediocre** | | **Good** | |  |
| **Locker /Locker room** | | Unclean, may not be well-lit, inadequate dressing space or receptacles provided, plumbing is almost unusable | Most areas are clean, lockers and/or dressing space provided (but is inadequate), plumbing could be improved, but works | | clean, well-lit, lockers and/or dressing space provided, plumbing works well | |
| **Bathrooms** | | Bathroom is not clean, not well-stocked. More than 50% of fixtures are in disrepair | Bathroom is fairly clean, stocked, and most sinks and toilets’ plumbing are in good working order. | Bathroom is clean, well-lit, stocked, all plumbing is functioning well. | |  |
| **Benches** – all types of affixed seating. | | Benches are in bad condition, unusable | Benches are missing some paint or boards, may be crooked, but otherwise usable | In good condition but could have minor cosmetic flaws | |  |
| **Trash containers (availability and adequacy)** | | Unclean and/or in poor condition, more care needed, Full with trash or overflowing.  Not adequate | Partially unclean or in < perfect condition, but scattered, and unstable  adequate | Clean on exterior, scattered throughout, not overflowing with trash  Adequate | |  |
| **Drinking water facility** | | Either all or most (50%) are broken | At least 1 of the total facilities not in working operation | Working, clean water facility with clean surrounding area | |  |
| **Lighting –**For an outdoor resource such as a park, this is within the boundaries | | Area has limited lighting, inadequate for safety | Areas has some lighting | Area or building has effective overhead lighting which is sufficient for safety | |  |
| **Shelters** – | | Structures are not intact – so rain would get into area. If seating/tables are present, they are in major need of repair or are missing | Structures are in need of some repair, provide protection from weather. If seating/tables are present they are usable but need minor repair | Structures are intact, provide protection from weather. If seating/tables are present they are clean. | |  |
| **Incivilities** | | **Little/few** | **Some** | **A lot** | |  |
| **Auditory annoyance** | | Sound is not irritating, but is (hardly) noticeable | Sound(s) is (are) noticeable and interfere(s) with enjoyment of resources | Noticeable sounds which are unpleasant. Reaction is to leave area. | |  |
| **Litter/ refuse** | | Litter present in the corners , not interfering the usability of the facility | Minimal Litter present along the usable part of the facility, not hindering the usability | Litter present in a quantity hindering the usability of the facility | |  |
| **Overgrown grass/ shrubs** | | A little bit, hardly noticeable | A moderate amount, noticeable | A lot, very noticeable, may be obstructing some equipment | |  |

1. **Capacity** = (for an indoor facility) The maximum capacity number which can perform activities at a time(ask the management)

2. **Hours of Operation** = The hour that the resource opens and closes (24 hour clock—hrs:mins; e.g. 05:30 A.M.= 0530 & 05:30 P.M.= 1730 )

3. **Features** --

Rate each item individually by circling a number according to the above stated Operational Definitions.

0 = Not Present 1 = Poor 2 = Mediocre 3 = Good

4. **Amenities --**

Assess for entire resource

Rate each item individually by circling a number according to the above stated Operational Definitions.

0 = Not Present 1 = Poor 2 = Mediocre 3 = Good

5. **For Incivilities**--

Assess for entire resource

Rate each item individually by circling a number according to the above stated Operational Definitions.

0 = Not Present 1 = Little/Few 2 = Some 3 = A lot

**Supplementary Table 2. Prevalence of insufficient physical activity by socio-demographic characteristics of the study population**

| **Socio-demographic characteristics** | **Insufficient physical activity n (%)** |
| --- | --- |
| **Age Group** |  |
| 18-29 (n=146) | 57 (39.0) |
| 30-39 (n=121) | 44 (36.4) |
| 40-49 (n=91) | 37 (40.7) |
| 50-59 (n=64) | 21 (32.8) |
| 60+ (n=72) | 29 (40.3) |
| **Gender** |  |
| Male (226) | 85 (37.6) |
| Female (268) | 103 (38.4) |
| **Education** |  |
| No formal schooling (n=86) | 30 (34.9) |
| Less than primary school (n=42) | 15 (35.7) |
| Primary school completed (n=28) | 11 (39.3) |
| Middle school completed (n=114) | 40 (35.1) |
| High school completed (n=76) | 22 (28.9) |
| PUC/ITI/Diploma (n=78) | 35 (44.9) |
| College/Degree completed (n=53) | 27 (50.9) |
| **Occupation** |  |
| Government employee (n=36) | 19 (52.8) |
| Non-Government employee (n=109) | 30 (27.5) |
| Self-employed (n=109) | 44 (40.4) |
| Student (n=30) | 13 (43.3) |
| Homemaker (n=184) | 74 (40.2) |
| Retired (n=19) | 7 (36.8) |
| Unemployed (n=7) | 1(14.3) |
| **Religion** |  |
| Hindu (n=286) | 91 (31.8) |
| Muslim (n=196) | 91 (46.4) |
| Christian (n=12) | 6 (50.0) |
| **Income quintile** |  |
| Lowest (n=80) | 15 (18.8) |
| Second (n=72) | 27 (37.5) |
| Middle (n=99) | 41 (41.4) |
| Fourth (n=46) | 23 (50.0) |
| Highest (n=195) | 80 (41.0) |

**Supplementary table 3. Distribution of resources for performing physical activity in Kolar city by nature of user fee collection**

| **Nature of Resources** | **Free** | **Pay at door** | **Pay for certain programmes** | **Others** | **Total** |
| --- | --- | --- | --- | --- | --- |
| Stadium | 1 | 0 | 1 | 0 | **2** |
| Park | 13 | 0 | 0 | 0 | **13** |
| Playground | 6 | 0 | 0 | 0 | **6** |
| Fitness centers | 0 | 13 | 0 | 0 | **13** |
| Yoga centers | 4 | 2 | 0 | 2 | **8** |
| Independent sports facility | 1 | 0 | 0 | 0 | **1** |
| Sports club | 10 | 2 | 0 | 1 | **13** |
| Total (N=56) | 35 (63.6%) | 16 (29.1%) | 1 (1.8%) | 3 (5.5%) | **56 (100%)** |
| **Pay at door**: Resources can only be accessed after payment at regular intervals  **Pay for certain programmes**: Resources are freely accessible but for utilizing few programmes one needs to pay.  **Others**: Other than above stated payment categories e.g. one time registration fee. | | | | | |
